# Serous borderline tumor of the fallopian tube presented as hematosalpinx: a case report

**DOI:** 10.1186/1471-2407-5-129

**Published:** 2005-10-07

**Authors:** Maja Krasevic, Teodora Stankovic, Oleg Petrovic, Neda Smiljan-Severinski

**Affiliations:** 1 Department of Pathology, School of Medicine, University of Rijeka, Br. Branchetta 20, Rijeka, Croatia; 2 Department of Citology, Clinical Hospital Centar, Rijeka, Cambierieva 17/5, Croatia; 3 Department of Obstetrics and Gynecology, Clinical Hospital Centar, School of Medicine, University of Rijeka, Cambierieva 17/5, Rijeka, Croatia

## Abstract

**Background:**

Compared with their ovarian counterparts, serous borderline tumors of the fallopian tube are uncommon, with limited experience about their clinical behaviour. We present a case of serous borderline tumor of the fallopian tube with unusual presentation and summarise all the published cases to date.

**Case presentation:**

A case of serous borderline tumor of the fallopian tube in a 34-year old patient is presented, incidentally found during routine gynecologic examination. At laparoscopy the tumor was unusualy presented as hematosalpinx and was treated by salpingectomy. Cell-cycle analysis of the tumor tissue revealed a diploid DNA content and a low S-phase fraction. There was no evidence of the disease during the follow-up period of 4.6 years.

**Conclusion:**

The current case and review of the literature suggest salpingectomy as the optimal treatment for patients with serous borderline tumor of the fallopian tube.

## Background

Compared with their ovarian counterparts, serous borderline tumors (SBTs) of the fallopian tube are uncommon, with limited experience about their clinical behaviour.

Herein we report a case of SBT of the fallopian tube in a young woman that was unusualy presented as hematosalpinx during the laparoscopic operation.

## Case presentation

A 34-year old multigravida was found to have right adnexal mass on her routine gynecologic examination. Her previous medical history was uneventfull and Pap smear was normal. Transvaginal ultrasonography identified a cystic mass adjacent to the right ovary. Serum CA 125 was 5.1 U/ml (reference range: < 35 U/ml). At laparoscopy a dilated fallopian tube with bluish discoloration was found. The contralateral fallopian tube, ovaries and uterus were unremarkable. Exploration of the abdomino-pelvic cavity revealed smooth and shiny peritoneal surphace. Obtained peritoneal and pelvic washing were negative. Fine needle aspiration of dilated part of the fallopian tube revealed a 4 ml of bloody content. Cytological findings were consistent with hematosalpinx. Right salpingectomy was performed without using endoscopic bag. The patient was followed up by means of ultrasonography and serum CA 125 for 4.6 years. During this period she had no evidence of the disease.

**Figure 1:**
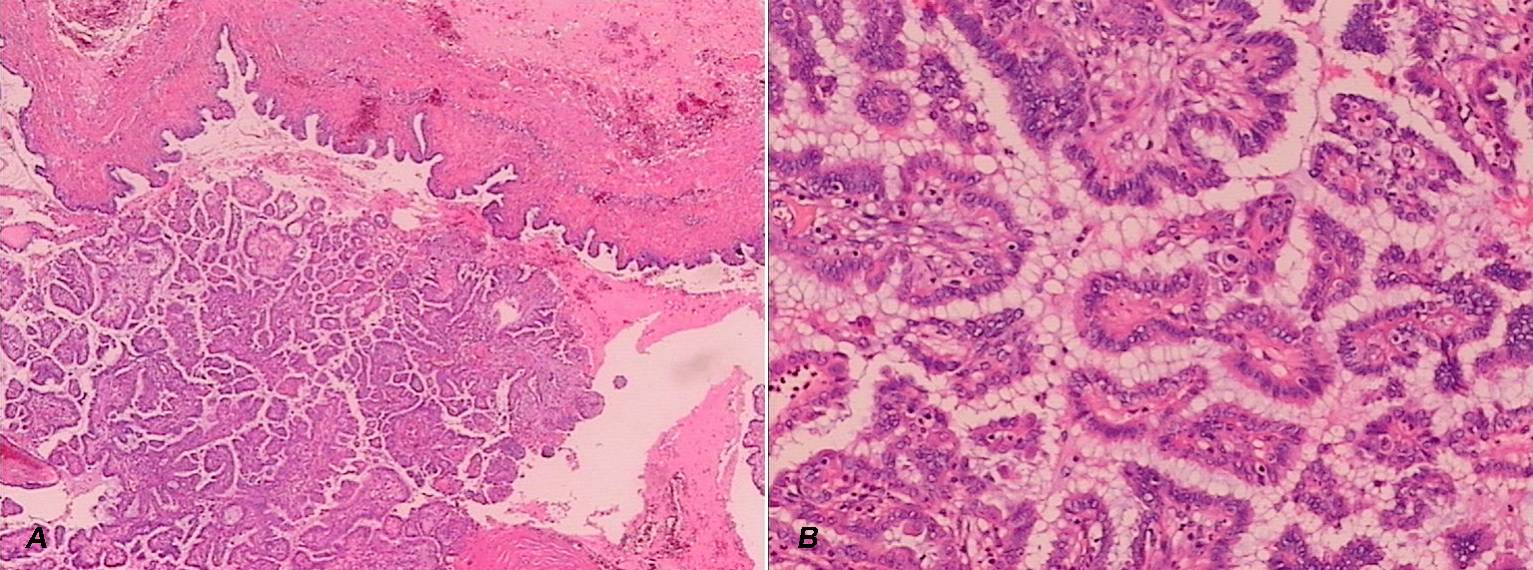
**Composite figure of serous borderline tumor of the fallopian tube**. **A) **Papillary tumor protruding into the lumen of the fallopian tube (low magnification). There is no invasion into the wall of the tube (top). **B) **Stratification and budding of the epithelium with focal nuclear atypia (high magnification).

Grossly, a 7.0 cm long fallopian tube was irregularly dilated up to 4.4 cm in diameter at the ampulary region. The fimbriae were intact. The serosal surphace was smooth. Sections of the dilated part of the fallopian tube revealed a cystic tumor with focally yellow to tan, soft papillary excrescences protruding into the lumen (Fig. 1A) and foci of intracystic hemorrhage. On microscopic examination the papillae were covered by serous type of epithelium, displaying stratification and budding with focal nuclear atypia (Fig. 1B). Three types of cells were recognised; ciliated cells, hob-nail cells and mesothelium-like cells. Small foci of tumor tissue necroses and hemorrhage were noted. There was no invasion of the supportive stroma of the papillae or into the fallopian tube wall. Focus of endosalpingiosis within the adjacent mesosalpinx was found. DNA analysis determined by flow cytometry paraffin technique revealed DNA diploid tumor with low S-phase fraction of 6.5 %.

## Conclusion

To the best of our knowledge, only 6 cases of SBTs of the fallopian tube have been previously reported in the literature. Clinico-pathologic features of these tumors, including the current case are summarized in Table 1. The first case [1] that was originally reported as adenocarcinoma, Alvaredo-Cabrero et al [2] considered it to be a tumor of borderline malignancy. Age of the patients ranged from 19 to 34 (average 29.2) years. Four tumors were found incidentaly [2-4], three were presented with abdominal pain [1,5,6]. Serum CA 125 was elevated in one case [5]. All the tumors were unilateral, measuring from 1.7 – 13 cm. Three of them were located at the fimbriated end, two at the ampulary region, and in one case the whole tube was enlarged. Grossly, all the tumors were cystic with internal papillation, except one that was solid [5]. Histologicaly the epithelium showed the morphology of borderline serous tumors, according to the criteria applied in the ovary.

SBTs were treated exclusively by surgery, most of them by unilateral salpingectomy. Complete staging/restaging was performed only in one case [5], where except salpingo-oophorectomy, contralateral ovarian biopsy, partial omentectomy and appendectomy were performed. Peritoneal and pelvic washing were obtained and multiple pelvic lymph nodes were sampled. Ten months later, contralateral ovary due to adenofibroma was removed and peritoneal and pelvic washing were obtained. The patient was without evidence of the disease during the follow-up period of 6 years.

Seidman and Kurman [7] reviewed 97 reports that included 4,129 patients with ovarian SBTs. They concluded that surgical-pathological stage and type of extraovarian disease (invasive versus noninvasive implants) were the most powerful prognostic indicators for these tumors. Survival rate for stage I SBTs was almost 100 % (99.5 %). Survival for advanced stage SBTs with noninvasive and invasive implants were 95.3 % and 66 % respectively.

In order to predict the behaviour of the SBT in current case, similary to the ovarian tumors, DNA ploidy was determined. In our case the tumor was diploid with low S-phase fraction of 6.3 %. DNA ploidy was found to be important prognostic factor in 321 patients with ovarian borderline tumors (BTs) [8]. Aneuploidy was associated with older age, more advanced disease and non-serous histologic type as well. However, although aneuploidy correlated with higher stage in a study of 42 ovarian BTs, ploidy/DNA index could not predict which borderline tumors would behave in a more aggressive fashion [9]. Analysis of 49 advanced stage ovarian SBTs revealed no correlation between DNA content and frequency of tumor recurrence [10]. No death occurred in a subset of patients with aneuploid tumors. The patients with aneuploid tumors were more likely to have received adjuvant chemotherapy, that could affect the results.

Four additional cases of BTs of the fallopian tube were reported. Three had mucinous type of epithelium [11-13] and one was endometrioid borderline tumour [2]. The mean age of patients with mucinous borderline tumors (MBT) was 54 years. Presented symptoms included constipation [11] and pelvic mass [11,13]. MBT was bilateral in one case [13]. Two cases were associated with pseudomyxoma peritonei [11,12]. Follow-up informations were not available in these cases.

The unique case of borderline endometrioid tumor was reported by Alvaredo-Cabrero et al in a serie of 20 cases of tumors of the fimbriated end of the fallopian tube [2]. The tumor was borderline adenofibroma and contained large number of endometrioid-type glands with focal villoglandular pattern separated by a fibromatous stroma. No follow-up information was available.

In conclusion, confinement to the tube as well as normal DNA content of the tumor may indicate a benign clinical behaviour of the SBTs of fallopian tube, suggesting unilateral salpingectomy as the optimal treatment for these young patients. However, long term follow-up and more cases are needed to make definitive conclusions.

## Competing interests

The authors declare that they have no competing interests.

## Authors' contributions

TS carried out the cytologic examination and participated in the design of the study.

OP and NSS have performed the operation and participated in drafting the manuscript.

MK carried out the pathohistological examination and participated in writing the manuscript.

All authors read and approved the final manuscript.

## Pre-publication history

The pre-publication history for this paper can be accessed here:



## Figures and Tables

**Table 1:** Serous borderline tumors of the fallopian tube: clinical and pathological features of 7 cases.

Case no. (reference no.)	Original diagnosis	Age (yr)	Symptoms	Procedure	Localization	Gross finding	Follow-up
1. (1)	papillary cystadenocarcinoma	19	lower-guadrant pain, coul-de-sac mass	partial salpingectomy	fimbriated end	4 cm, cystic with papillations	18 mo, WED
2. (3)	borderline cystadenofibroma	32	IF during an early pregnancy	salpingectomy	ampulary region	2.5 cm, cystic with papillations	WED after unspecified time
3. (5)	borderline papillary serous tumor	31	abdominal pain, elevated CA 125	salpingo-oophorectomy, ovarian biopsy, partial omentectomy	protruding from the fimbria	6 cm, solid polipoid mass with cauliflower-like surface	6 yr, WED
4. (2)	borderline serous tumor	NS	IF during an elective operation	NS	fimbriated end	1.7 cm, cystic with papillations	2.4 yr, well
5. (4)	serous tumor of low malignant potential	31	IF on routine gynecologic examination	salpingectomy	entire tube	13 cm, cystic with papillations	not available
6. (6)	serous tumor of low malignant potential	28	lower quadrant pain	salpingectomy	NS	5 cm, cystic with papillations	recently found
7. (current case)	serous tumor of borderline malignancy	34	IF on routine gynecologic examination	salpingectomy	ampulary region	4.4 cm, cystic with papillations	4.6 yr, WED

NS, not specified; IF, incidental finding; WED, without evidence of the disease.
